# Genes Responsible for H_2_S Production and Metabolism Are Involved in Learning and Memory in *Drosophila melanogaster*

**DOI:** 10.3390/biom12060751

**Published:** 2022-05-26

**Authors:** Olga G. Zatsepina, Lyubov N. Chuvakova, Ekaterina A. Nikitina, Alexander P. Rezvykh, Alexey S. Zakluta, Svetlana V. Sarantseva, Nina V. Surina, Alexander L. Ksenofontov, Ludmila A. Baratova, Viktoria Y. Shilova, Michael B. Evgen’ev

**Affiliations:** 1 Engelhardt Institute of Molecular Biology, Russian Academy of Sciences, 119991 Moscow, Russia; olzacepina@yandex.ru (O.G.Z.); lyubov.astakhova@gmail.com (L.N.C.); aprezvykh@yandex.ru (A.P.R.); herbariumcat@gmail.com (A.S.Z.); vika-shilova@yandex.ru (V.Y.S.); 2 Department of Neurogenetics, Pavlov Institute of Physiology, 199034 Saint Petersburg, Russia; 21074@mail.ru; 3 Department of Human and Animal Anatomy and Physiology, Herzen State Pedagogical University, 191186 Saint Petersburg, Russia; 4 Petersburg Institute of Nuclear Physics, Russian Academy of Sciences, 188300 Gatchina, Russia; svesar1@yandex.ru (S.V.S.); anilannas123@gmail.com (N.V.S.); 5 Belozersky Institute of Physico-Chemical Biology, Lomonosov Moscow State University, 119991 Moscow, Russia; alexksenofon@gmail.com (A.L.K.); baratova@belozersky.msu.ru (L.A.B.)

**Keywords:** *Drosophila melanogaster*, CBS, CSE, H_2_S, learning, memory, courtship rejection paradigm, transcriptome

## Abstract

The gasotransmitter hydrogen sulfide (H_2_S) produced by the transsulfuration pathway (TSP) is an important biological mediator, involved in many physiological and pathological processes in multiple higher organisms, including humans. Cystathionine-β-synthase (CBS) and cystathionine-γ-lyase (CSE) enzymes play a central role in H_2_S production and metabolism. Here, we investigated the role of H_2_S in learning and memory processes by exploring several *Drosophila melanogaster* strains with single and double deletions of CBS and CSE developed by the CRISPR/Cas9 technique. We monitored the learning and memory parameters of these strains using the mating rejection courtship paradigm and demonstrated that the deletion of the *CBS* gene, which is expressed predominantly in the central nervous system, and double deletions completely block short- and long-term memory formation in fruit flies. On the other hand, the flies with *CSE* deletion preserve short- and long-term memory but fail to exhibit long-term memory retention. Transcriptome profiling of the heads of the males from the strains with deletions in *Gene Ontology* terms revealed a strong down-regulation of many genes involved in learning and memory, reproductive behavior, cognition, and the oxidation–reduction process in all strains with *CBS* deletion, indicating an important role of the hydrogen sulfide production in these vital processes.

## 1. Introduction

Various roles of the third described endogenous gasotransmitter (H_2_S) under normal conditions and in various human diseases and pathologies were described in several excellent reviews [1,2,3]. Similarly, the transsulfuration pathway (TSP), which results in the production of hydrogen sulfide (H_2_S) and includes the conversion of homocysteine to cysteine following the breakdown of methionine, was described in detail by several authors including a brilliant analysis performed by Kimura [4,5,6,7]. Briefly, the accumulated data demonstrate that, in Drosophila and other eukaryotes, H_2_S is produced by cystathionine β-synthase (*CBS*), cystathionine γ-lyase (*CSE*), and 3-mercaptopyruvate sulfurtransferase (*MST*) genes that have different functions and may be expressed in different organs and tissues [8]. It becomes clear that H_2_S plays an important role in brain functions as a neuroprotector and neuromodulator in different organisms, including humans. There are several lines of evidence in favor of this conclusion. First, the level of endogenous H_2_S is significantly decreased throughout aging and in the case of many neurodegenerative diseases including Alzheimer’s Disease (AD) [9,10]. Second, the application of various H_2_S donors often exhibited a strong neuroprotective effect, and such treatment may ameliorate memory impairment and restore cognitive functions in various model studies and the case of several human neurodegenerative diseases [11,12,13,14]. Finally, numerous missing mutations that disrupt the structure of CBS in humans result in classical homocystinuria due to cystathionine β-synthase (*CBS*) deficiency [15,16]. Individuals with homocystinuria, the most frequent disorder of sulfur metabolism, have many developmental and cognitive difficulties, with a significant number of cases having a learning disability, atherosclerosis, or thromboembolic disease [15,17]. A decrease in endogenous H_2_S generation contributes to homocysteine (Hcy)-induced deficit in learning and memory in rats [18]. In contrast, exogenous H_2_S ameliorated Hcy-induced cognitive dysfunction [19]. Interestingly, the vision phenotype of CBS knock-down flies is consistent with severe myopia observed in homocystinuria patients [20].

However, to the best of our knowledge, there are only scattered data implicating the synthesis of endogenous H_2_S in the memory process and mating behavior under normal conditions in higher organisms [21,22]. *Drosophila melanogaster* strains obtained in our laboratory [23] containing single and double deletions of the three major genes implicated in H_2_S production represent a convenient model to study the role of H_2_S production in learning and memory processes in higher organisms.

We hypothesized that the disturbance of endogenous H_2_S generation and metabolism in the brain may affect cognitive functions in *Drosophila melanogaster*, a model organism often used in the investigations of molecular mechanisms underlying learning, memory, and aging in higher eukaryotes [24,25,26,27].

In the experiments described herein, we made use of several *D. melanogaster* strains obtained using the CRISPR/Cas9 technique. These strains with deletions of the three genes (*CBS, CSE,* and *MST*) were used and described in detail in our previous studies [23,28]. Transcriptomic studies demonstrated that the deletions of both *CBS* and *CSE* (*CBS-/-*; *CSE-/-*) have a cumulative effect and drastically alter genome expression, with a more pronounced impact exerted by the deletion of the *CBS* gene. The previous analysis demonstrated that the obtained deletions of *CBS* and *CSE* affected numerous genes involved in various biological pathways including oxidation-reduction process, glutathione metabolic process, stress-response genes, housekeeping genes, and genes participating in olfactory and reproduction, while the deletion of *MST* affected a comparatively small number of genes [23]. Based on the above-mentioned facts and considerations, we decided to monitor the learning and memory parameters of these strains (*CBS-/-* and *CSE-/-*), as well as strains with deletions of both *CBS* and *CSE* genes (*CBS-/-; CSE-/-*) using the mating rejection courtship paradigm. Our studies demonstrated that the deletion of the *CBS* gene and double deletions completely block short- and long-term memory formation in *D. melanogaster* males, while the flies with *CSE* deletion preserve short- and long-term memory but failed to exhibit long-term memory retention. Transcriptomic studies revealed a pronounced down-regulation in the expression of multiple pertinent genes and signal pathways in the heads of the male flies from the studied strains with deletions of major H_2_S-producing genes.

## 2. Materials and Methods

### 2.1. Fly Strains and Maintenance

All flies were reared on a standard sugar–yeast–agar medium at 25 °C, 60% humidity, and a light–dark cycle of 12:12 h. As a control, we used strain 58492 with genotype (y1 M{Act5C-Cas9.P. RFP-}ZH-2A w1118 DNAlig4169) obtained from the Bloomington Drosophila Stock Center. Additionally, we used transgenic *CBS-/-*(5) and *CBS-/-*(8), *CSE-/-*, (*CBS-/-*; *CSE-/-*(1)) and *(CBS-/-*; *CSE-/-*(2)) strains developed in our laboratory [23].

### 2.2. Test for Learning and Memory of Flies in Conditioned Courtship Suppression Paradigm

To evaluate memory formation in drosophila males, we used the conditioned courtship suppression paradigm (CCSP) [29]. *Drosophila melanogaster* males drastically reduce courtship behavior after mating failure. Under laboratory conditions, such conditioned courtship suppression serves as a complex learning and memory assay. Interestingly, variations in the courtship conditioning assay can establish different types of memory [27,30,31]. CCSP is used widely for learning ability and memory retention in Drosophila [32,33,34]. All procedures were performed exactly as described in our previous paper [35].

Briefly, the resulting courtship index [36], i.e., percentage of time spent in courtship over a 300-s period) was calculated for each male [36]. The CI was used to calculate the learning index (LI) as follows: LI = ({CI_na_ − CI_tr_}/CI_na_) × 100 = (1 − CI_tr_/CI_na_) × 100 [37], where CI_na_ and CI_tr_ are the mean courtship indices for independent samples of naïve and trained males, respectively.

Statistical comparisons of behavioral data were performed using a two-sided randomization test [38] by directly computing the probability of rejection of the null hypothesis α_R_. The sampled randomization test with 10,000 permutations was used. The null hypothesis was rejected at α_R_ < 0.05. We compared all experimental groups with each other.

### 2.3. Olfactory Behavior Assay

Olfactory behavior assays were performed in a temperature-controlled darkroom at +25 °C. For experiments, we used a classical T-maze [39]. Two airflows were supplied to tubes of T-maze. The first airflow carried the odorant (4-methyl cyclohexanol, Fluka, Sigma-Aldrich, Germany) dissolved in mineral oil (Vecton, Russia) with 1:100 dilution, another one carried fresh air passed through mineral oil without odorant. The concentration of odorant was chosen based on the previous investigation [40] to ensure strong repulsive behavior in the control flies. About fifteen 5-day-old naïve males were transferred into a T-maze central camera for 2 min for adaptation without any airflow, and then the test was performed for 3 min for a choice between airflows with or without the odorant. Thereafter, flies in the odorant-contained tube were counted (*N_odor_*) and the percentage (*V*) of flies that were attracted (non-repulsed) by the odorant was counted:

$$V\left(\%\right)=\frac{N_{odor}\times 100\%}{N_{total}}$$

The experiment included 31 replicates and about 400 flies participated in the assay for each genotype. Statistical analysis was performed using KyPlot 5.0 software (KyensLab Inc., Tokyo, Japan, software 5.0). All samples were tested for normality with the Shapiro–Wilk test which suggested that samples have non-normal distribution, so the Mann–Whitney test was applied to compare each experimental sample with the control one.

### 2.4. RNA Extraction from Fly Heads for Library Preparations

Three biological replicates of 5-day-old naïve males were collected for each transcriptome analysis. For each replicate, total RNA was isolated from approximately 100 heads of naïve males collected by decapitation of drosophila males on an ice-cooled surface. The extraction was made using RNAzol RT (Molecular Research Center, Cincinnati, OH, USA) according to the company’s protocol. The concentration and quality of RNA were determined via Qubit Fluorometer (Invitrogen) and an Agilent BioAnalyzer 2100, respectively, using RNA 6000 nano kit (Agilent technologies, Santa-Clara, CA, USA). For each of the three biological replicates taken for libraries, 100 heads were collected. For libraries RNA with Integrity Number (RIN), no less than 8 were taken.

### 2.5. cDNA Library Preparation and Data Analysis

Illumina NEB Next Ultra II Directional RNA Library Prep Kit (NEB, Ipswich, MA, USA) was used for mRNA libraries preparation. The sequencing was performed on the Illumina NextSeq 500 platform. For each library sequencing provided around 15–20 million reads. For analysis, the PPLine script [41] was used: the reads were mapped to the *D. melanogaster* genome (Dm6) with STAR [42] following adapter, length, and quality trimming by Trimmomatic [43]. The edgeR package [44] was used for differential gene expression analysis. Differential expression parameters for each gene were estimated using TMM read normalization method, with the fitting quasi-likelihood negative binomial generalized log-linear model. Raw *p*-values were corrected with the FDR method. The topGO (v.2.36.0) and clusterProfiler Bioconductor packages [45] were used to perform Gene Ontology and KEGG enrichment analyses. Visualization of the gene set enrichment analysis (GSEA) was performed using custom scripts written in Python and R. Sequence data were deposited in the NCBI GEO database under the number—GSE200397. RNA sequencing and further differential expression estimation were performed using the equipment of the Engelhardt Institute of Molecular Biology RAS “Genome” center (http://www.eimb.ru/rus/ckp/ccu_genome_c.php, accessed on 22 February 2022).

### 2.6. Quantitative Real-Time PCR

For real PCR, RNA was isolated from three biological replicates (fly heads). One microgram of total RNA was used for cDNA synthesis with an MMLV RT kit (Evrogen, Moscow, Russia, cat# SK021). All qRT-PCR reactions were conducted using the SYBR Green fluorescent dye (Evrogen, Moscow, Russia, cat.# PK156S) in an ABI PRISM VR 7500 device (Applied Biosystems). The relative expression of the studied genes was calculated based on the ΔΔCt method [46]. Quantifications were normalized to the housekeeping gene *rp49* [47]. Experiments were performed with three replicates and three experimental replicates. The primers sequences are listed in Table S1.

### 2.7. Amino Acid Quantitative Analysis

Intracellular metabolites from total flies were extracted using cold 80% (*v*/*v*) aqueous methanol [48]. The profile of amino acids was analyzed during separation in a lithium buffer system. Amino acids were quantified according to [49] using an L-8800 amino acid analyzer (Hitachi Ltd., Tokyo, Japan). For high-performance liquid chromatography, the 2622SC-PF ion-exchange column (Hitachi Ltd., P/N 855–4507, 4.6 mm × 60 mm, Tokyo, Japan) was eluted at a rate of 0.35 mL/min by step gradients of Li–citrate buffers and temperature (in the range 30–70 °C).

## 3. Results

### 3.1. Learning and Memory of Flies in Conditioned Courtship Suppression Paradigm

Courtship behavior is an important aspect of mating success, and many animals have intricate behavioral rituals that they use to attract mates. Thus, males of *D. melanogaster* have a highly stereotyped courtship routine that involves orientation towards the female, vibrating an outstretched wing to produce a courting song, licking, and attempted copulation. Courtship conditioning involves a training period, where learning and memory are induced, followed by a testing period where the behavioral effects of training are observed over time [30]. Courtship conditioning can be used to induce different temporal forms of memory, such as short- and long-term memory (STM and LTM).

**Figure 1:**
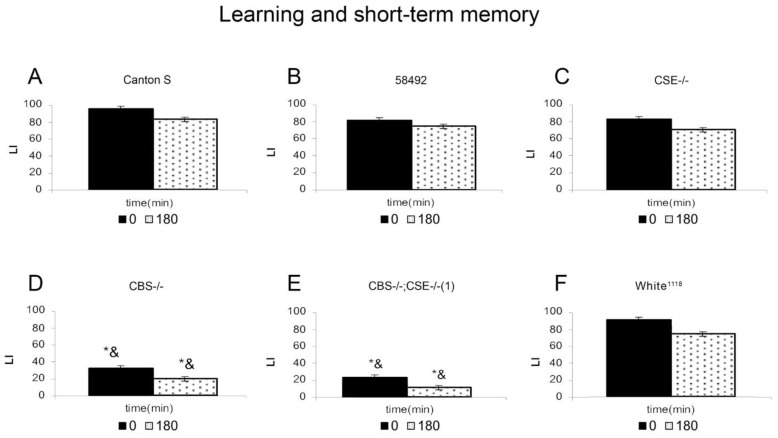
Dynamics of learning acquisition and short-term memory retention of conditioned courtship suppression in mutant males. Males from *Canton-S* (**A**), 58492 (**B**), *CSE-/-* (**C**), *CBS-/-* (**D**), *CBS-/-*; *CSE-/-* (1) (**E**), and *white*^1118^ (**F**) strains were tested. Abscissa: time after training (min); ordinate: LI—learning index, standard units. The sample size for each time point was 20 males. *—LI significantly lower than that 58492 strain under similar conditions (two-sided randomization test, α_R_ < 0.05); &—LI significantly lower than that of wild type *Canton-S* strain under similar conditions (two-sided randomization test, α_R_ < 0.05).

Figure 1 shows the results of the short-term memory analysis. To check the influence of differences in the genetic background on memory formation in the mating rejection paradigm, we used three strains as controls. These are the wild-type *Canton-S* (*CS*) strain, strain 58492, which, like the transformants, carries mutations in the yellow and white genes, and the white-eyed strain *white*^1118^. Males from wild-type *CS*, 58492, *white*^1118^ and *CSE-/-* are capable of learning and forming short-term memory. In contrast, males from strains *CBS-/-* and (*CBS-/-; CSE-/-*) (double deletion) exhibited very low ability to learn and, therefore, to form short-term memory. These mutant males showed learning ability that was four-fold lower than that of *CS* flies. This fact demonstrates the involvement of sulfur metabolism genes in the learning process and the formation of short-term memory. It is noteworthy, that in the case of single deletions, we observe different results. Thus, *CSE-/-* males showed excellent learning and short memory formation and did not differ in this respect from males of all control strains. Characteristically, *CBS-/-* males are characterized by an inability to learn and form short-term memory, like double deletion flies. This indicates the involvement of cystathionine β-synthase expression in learning and memory processes.

**Figure 2:**
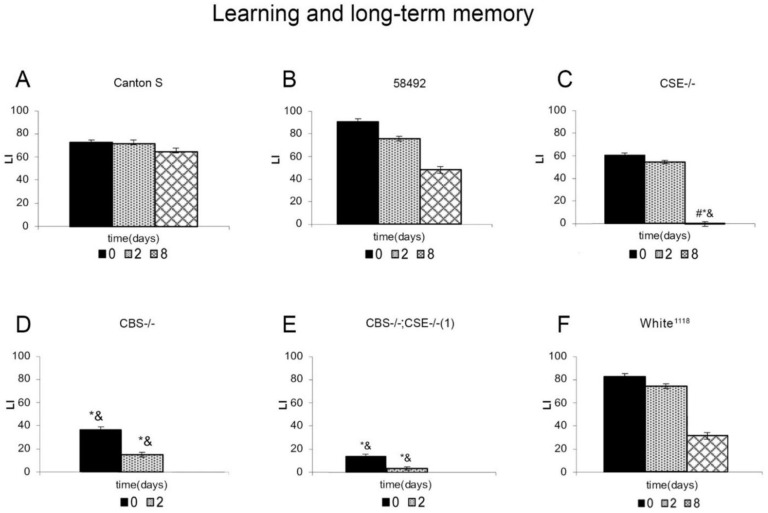
Dynamics of learning acquisition and long-term memory retention of conditioned courtship suppression in mutant males. Males from *Canton-S* (**A**), 58492 (**B**), *CSE-/-* (**C**), *CBS-/-* (**D**), *CBS-/-*; *CSE-/-* (1) (**E**), and *white*^1118^ (**F**) strains were tested. Abscissa: time after training (days); ordinate: LI—learning index, standard units. The sample size for each time point was 20 males. *—LI significantly lower than that 58492 strain under similar conditions (two-sided randomization test, α_R_ < 0.05); &—LI significantly lower than that of wild type *Canton-S* strain under similar conditions (two-sided randomization test, α_R_ < 0.05); and #—LI in the delayed test significantly lower than in test immediately following training (two-sided randomization test, α_R_ < 0.05).

In the next stage, we performed an analysis of learning acquisition and long-term memory retention in the studied strains (Figure 2). As expected, males from all control strains exhibited normal learning and long-term memory ability as the duration of learning increased, which is consistent with previous results [35,50]. However, in the case of males from white-eyed strains 58492 and *white*^1118^, a slight decrease in memory retention was observed. The results obtained in the analysis of learning acquisition and long-term memory retention in the strains with double deletion *CBS-/-*; *CSE-/-* are consistent with those of short-term memory; these males are incapable of learning and long-term memory formation. Thus, the presence of a double deletion leads to a disturbance of the ability to learn and form both short- and long-term memory.

Flies with *CSE* gene deletion exhibit a very peculiar behavioral pattern in our studies. In this case, learning indices immediately and after 2 days after training remain at a high level and do not significantly differ from those of the control strains, which indicates a normal implementation of learning processes and formation of long-term memory. However, after eight days, the learning index in this strain dropped catastrophically. Therefore, the absence of the cystathionine γ-lyase gene (*CSE*) does not affect the ability to learn and form short- and long-term memory but disrupts the retention of the latter, which in the wild type males lasts for up to 9 days. On the other hand, the *CBS* mutants, like the flies carrying the double deletion, turned out to be incapable of learning and long-term memory formation. Therefore, the ability to learn, regardless of the duration of the training, and the formation of short- and long-term memory, is severely impaired both in the absence of both genes (*CBS-/-*; *CSE-/-*) and in the case when only *CBS* gene is deleted.

All behavioral experiments investigating the mating rejection paradigm described above are sensible only if the flies being tested have normal olfactory abilities. To be sure that the males with deletions retain normal olfactory abilities, we performed a special series of experiments described below.

### 3.2. The Monitoring of Olfactory Abilities of the Strains Used in the Study

Our previous work dealing with transcriptome analysis of whole flies with the deletions of the three sulfur metabolism genes (*CBS*, *CSE*, and *MST*) revealed a decreased expression of several genes associated with response to pheromone [23]. Therefore, we decided to investigate the olfactory behavior of our strains with deletions as described in the Materials and Methods section. Our results demonstrated that all the experimental strains have the same repulsive behavior in response to high odorant concentration as the control ones (Figure S1).

### 3.3. Transcriptome Analysis of the Strains with Deletions

**Figure 3:**
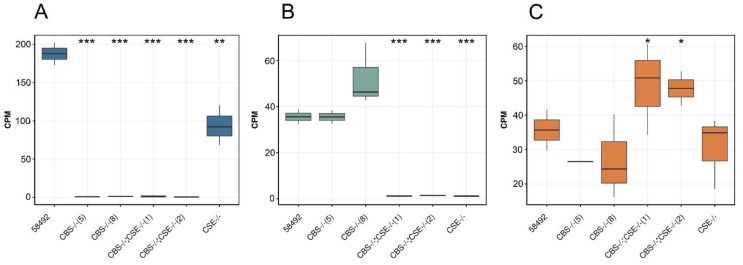
The expression of H_2_S producing genes in the heads of the males from the studied strains. (**A**) Box plot, demonstrating the expression levels of *CBS* in the heads of the flies from the studied strains. (**B**) Box plot, demonstrating the expression levels of *CSE* in the heads of the flies from the studied strains. (**C**) Boxplot, demonstrating the expression levels of *MST* in the heads of the flies from the studied strains. (* represents *p* < 0.05, ** represents *p* < 0.005, and *** denotes FDR < 0.001).

The performed analysis of the transcriptomic libraries obtained from the heads of the flies with deletions and control flies demonstrated that the expression of *CBS* in the heads of the control males from strain 58492 is 4–5 fold higher than the expression of *CSE* or *MST* (Figure 3). Surprisingly, in the strains with *CBS* deletion, we did not observe a compensatory increase in *MST* gene expression, which also produces hydrogen sulfide in the brain [51]. (Figure 3). The absence of a significant compensatory increase in the expression level of the *MST* gene in the strains with *CBS* deletion was confirmed by real-time PCR (Figure S2).

It is of note, that a performed amino acid quantitative analysis (see Materials and Methods) demonstrated (Figure S3) that, as expected, the deletion of the *CBS* gene resulted in the accumulation of homocysteine (Hcy) which behaves as a strong excitotoxic neurotransmitter that causes cognitive impairments, vascular dementia, and many other abnormalities in humans and mice [15,17,52]. The level of methionine/homocysteine mixture increases about 8-fold in *CBS-/-* strain and 7-fold in *CBS-/-*; *CSE-/-* strain. At the same time, the cystathionine level in the *CSE-/-* strain increases 170-fold. However, cystathionine accumulation detected in the case of *CSE* deletion (Figure S3) should have no deleterious effect because cystathioninemia is considered a biochemical abnormality without visible clinical symptoms [53].

**Figure 4:**
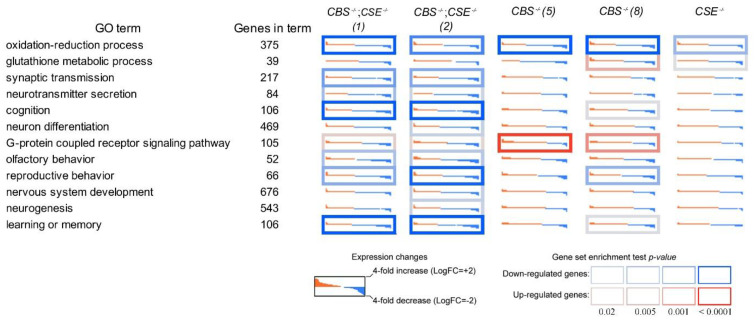
Gene ontology (GO) and pathway analysis of differentially expressed genes in the heads of naïve males. Expression level changes induced in the strains with single *CBS-/-*(5) and *CBS-/-*(8)*, CSE-/-* and double deletions *CBS-/-*; *CSE-/-*(1), and *CBS-/-*; *CSE-/-*(2) in the male heads. Each cell represents the sorted binary logarithms of expression value fold changes (LogFC) in the mutant strains versus control species for genes participating in a specific GO pathway. LogFC (vertical axis) ranges from -2 to +2, i.e., -2 from a 4-fold decrease (blue) to a 4-fold increase (red). Cell borders demonstrate the statistical significance of gene set enrichment analysis (Fisher test *p*-value): blue (enriched with downregulated genes) and red (enriched with overexpressed ones). (Color figure online). (For interpretation of the references to color in this figure legend, the reader is referred to the Web version of this article.).

The performed transcriptome analysis of libraries obtained from drosophila male heads of the strains with the deletions and a control 58492 strain enabled us to determine major cellular processes affected by the deletion of the two major genes involved in the transsulfuration pathway and H_2_S production. Thus, transcriptome profiling of the heads from the strains with deletions in *Gene Ontology* terms revealed significant changes in various cellular processes, including learning and memory, cognition, synaptic transmission, neurotransmitter transport, and secretion. Importantly, the performed analysis revealed a significant decrease in many genes involved in oxidation—reduction process, memory formation, and consolidation in the strains with the deletions. Characteristically, the most pronounced changes were revealed in the strains with double deletions (*CBS-/-*; *CSE-/-*) and the least in the strain comprising a deletion of the *CSE* gene (Figure 4).

The performed KEGG analysis of transcriptomic data from *Drosophila* heads (Figure S4) is discussed in the Supplementary Material.

For quantitative analysis of our transcriptomic data (to minimize unique single strain differences) at the first stage we picked up all genes that exhibited a similar pattern of expression changes: (1) in both strains with *CBS* deletions, (2) in both strains with double deletion (*CBS-/-*; *CSE-/-*) (Table 1).

**Figure 5:**
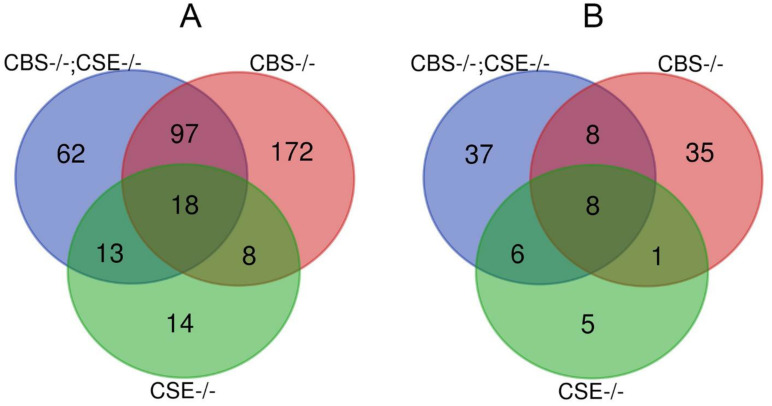
Venn diagram showing common and unique sets of differentially expressed genes between investigated strains (*CBS-/-*; *CSE-/-: blue*, *CBS-/-: red*, and *CSE-/-: green)* after pairwise comparison to control strain 548492 (FDR < 0.05); (**A**) Down-regulated genes. (**B**) Up-regulated genes.

Based on these data, Venn diagrams were made (Figure 5). It is evident from Figure 5 and Table 1 that the maximal number of genes that significantly changed their expression was revealed in the strains comprising the deletion of the *CBS* gene. Surprisingly, the strains with double deletion (*CBS-/-*; *CSE-/-*) exhibited a significantly smaller number of genes with expression changes in comparison with *CBS* strains (Table 1), while the strain with *CSE* deletion is characterized by a minimal number of genes with the altered expression.

It is necessary to emphasize that, in most cases, the genes with altered expression exhibited down-regulation. The group of down-regulated genes predominantly consists of the genes found in the strains with *CBS* deletion and the strains with double deletion (*CBS-/-*; *CSE-/-*) (Figure 5). Characteristically, most (70–75%) of the genes with altered expression in *CSE-/-* strain exhibited a similar pattern of expression changes in *CBS-/-* or (*CBS-/-*; *CSE-/-*) strains (Figure 5). In Table S2 (genes down-regulated) and Table S3 (genes up-regulated), we provide a list of all the genes with altered expression quantified in (Figure 5) with FDR < 0.05.

**Figure 6:**
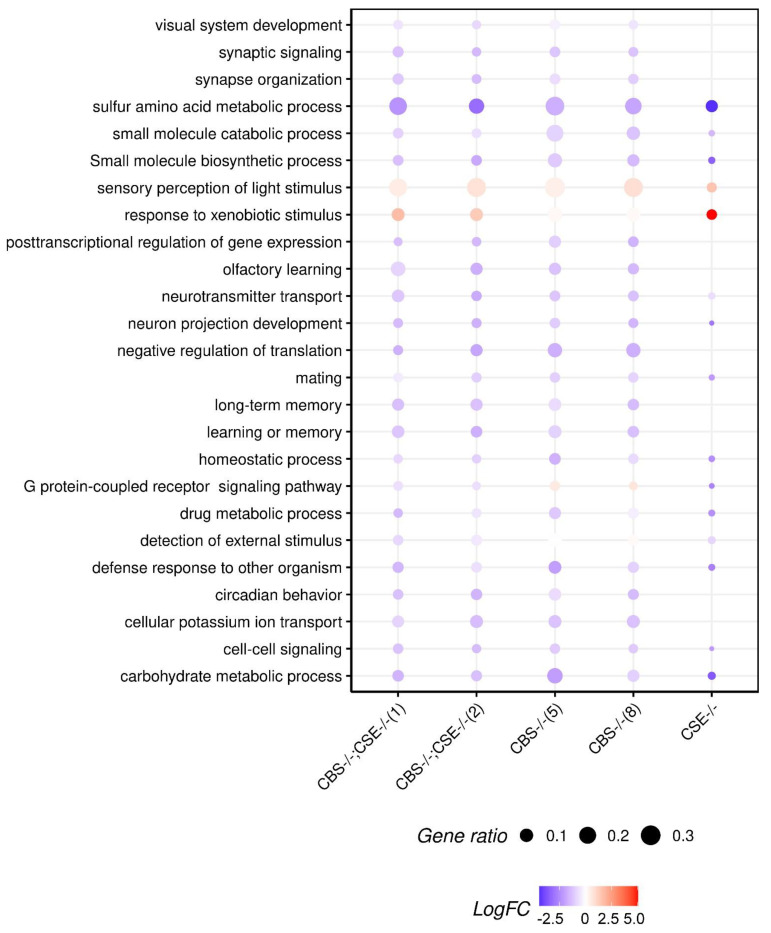
GSEA analysis of differentially expressed genes: *X*-axis represents strains, *Y*-axis represents, enriched Gene Ontology terms. Point colors represent averaged LogFC value between all genes (FDR < 0.05) appearing in the corresponding Gene Ontology group on the *Y* axis, point size—the proportion of genes with altered expression to the total number of genes in such group.

Additionally, we have performed a GSEA analysis of differentially expressed genes to reveal the gene categories that exhibited significant changes in their expression in the strains with deletions (Figure 6).

It is clear that GO terms related to the functioning of the nervous system and memory formation are of the greatest interest to our analysis. Figure 6 illustrates drastic changes in several major processes related to memory formation and consolidation observed in the strains with *CBS* deletion. These GO terms include learning and memory, short-term memory, long-term memory, cognition, synaptic signaling, synapse organization, neuron projection development, neurotransmitter transport, olfactory learning, cell–cell signaling, regulation of trans-synaptic signaling, synaptic vesicle localization, and vesicle-mediated transport in the synapse. It is of note that, in the strain with *CSE* deletion, significant changes (FDR < 0.05) in the expression of genes belonging to these groups are either absent or very small.

**Figure 7:**
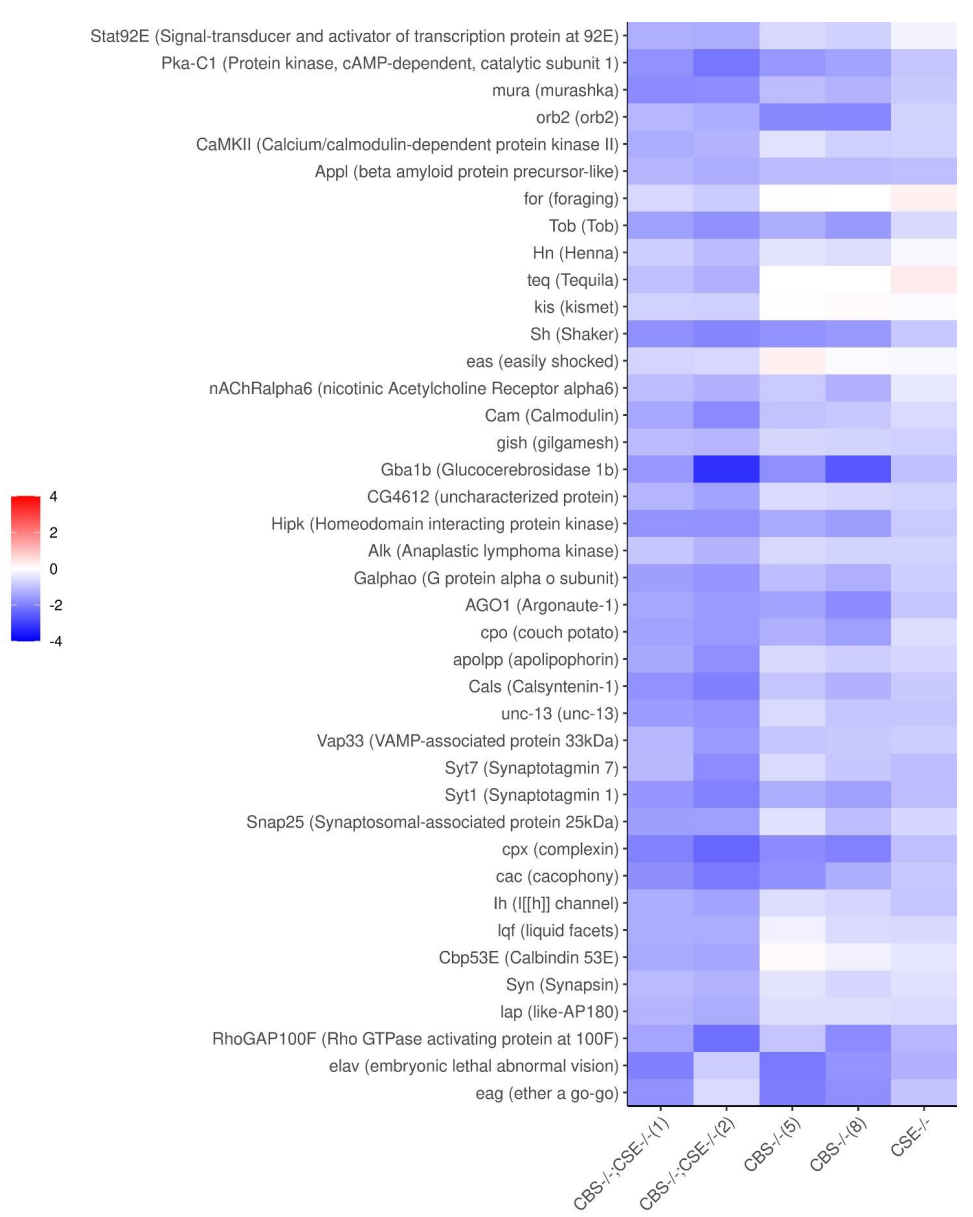
Heat map for down-regulated genes involved in learning and memory, cognition, synapse organization, and signaling in the strains with deletions. (Genes with FDR < 0.05 in at least one comparison were considered significant). All comparisons were with the control strain (58492).

To better illustrate the changes in pertinent gene expression in the studied strains we provided a heat map of the genes involved in learning and memory, cognition, synapse organization, and signaling (Figure 7). The heat map demonstrated that the *CSE* strain exhibited a slight, statistically insignificant decrease in the expression of almost all genes that belong to the down-regulation group in strains with *CBS* deletion. Importantly, the most drastic decrease in the expression of these genes is observed in both strains with double deletion.

Several genes with altered expression revealed by transcriptome analysis (i.e., *glucosidase* *beta* *acid* (*GBA1*) gene, *Tequila* (*Teq*) gene, and *Argonaute-1* (*Ago1*)) were studied using quantitative real-time PCR. It is evident that the most pronounced decrease in the level of expression in all strains with *CBS* gene deletion is observed for the *GBA1b* gene. Previously, using the courtship test, it was shown that Drosophila *GBA1b* mutants lack long-term memory [54]. The expression of the *Tequila* (*Teq*) gene, which encodes a serine protease (an orthologue of the human neurotrophin gene), is also necessary for long-term memory formation [55]. *Ago1* plays a role in miRNA-mediated translational control of neurons during LTM [56]. It shows a slight decrease in the expression level in all strains with *CBS* gene deletion. The expression of the *Teq* gene is downregulated only in (*CBS-/-*; *CSE-/-*) strains. Interestingly, *Rutabaga* (*rut*) gene, which encodes Ca [2+]/calmodulin-activated adenylyl cyclase that is responsible for synthesis of cAMP and needed in learning and memory [57], did not change its expression in our strains with deletions. The results of quantitative real-time PCR are depicted in Figure S5.

To better illustrate the relationship between genes with reduced expression levels and GO terms related to memory formation processes, a network was created for the (*CBS-/-*; *CSE-/-*(1)) strain. The picture demonstrates the complex relationships between such genes and the involvement of the same genes in different processes responsible for memory acquisition and consolidation (Figure S6).

## 4. Discussion

The physiological role of hydrogen sulfide was described in detail for the first time in the classical studies of Kimura in 1996 [4]. It has been also demonstrated in several investigations that H_2_S functions as a signaling molecule in the central nervous system (CNS), being involved in the regulation of ion channels, neurotransmitter functions, and various signaling molecules such as tyrosine kinases [4,58,59]. It has been established that CBS is the predominant H_2_S synthetase in CNS in all higher organisms [4,58,59,60]. Thus, neuronal localization of CBS protein was demonstrated in all major areas of the brain [59], in radial glia/astrocyte lineage of developing mouse brain [61,62], and in reactive astrocytes [63]. In contrast, the level of the other major H_2_S synthetase (CSE) and its activity is comparatively low in the mammalian brain [64].

In our studies presented here, we made use of several *D. melanogaster* strains developed in our laboratory exploring the CRISPR/Cas9 technique [23]. These strains containing single and double deletions of major genes responsible for H_2_S production represent a unique tool to investigate the role of endogenous H_2_S in learning and memory.

Previous numerous experiments demonstrated a strong protective role of exogenous H_2_S treatment in various pathologies, including neurodegenerative diseases and aging [65,66]. In addition, human mutations of *CBS* genes result in homocystinuria accompanied by strong cognitive impairments and many other abnormalities [16,18,19]. These data led us to hypothesize that the obtained deletions of *CBS* and *CSE* genes in Drosophila may be a perfect tool to reveal the role of individual H_2_S-producing genes in learning and memory processes.

Experiments have shown that strains with CBS deletions are characterized by a complete absence of short- and long-term memory, as shown in the courtship suppression paradigm (Figure 1 and Figure 2). Moreover, transcriptome studies using RNA isolated from the male heads demonstrated that all strains with *CBS* deletion exhibited a dramatic decrease in the expression of many genes involved in memory formation processes (Figure 6 and Figure 7). We are well aware that, in all strains containing a deletion of the *CBS* gene, the accumulation of toxic neurotransmitter homocysteine may influence the functioning of the nervous system and be at least partially responsible for the observed learning and memory impairments. However, here we demonstrated that while strains with double deletions and strains comprising only *CBS* deletion are characterized by a very similar level of *Hcy* (Figure S2), the most drastic drop of most pertinent gene expression was detected in the strains with double deletion (Figure 7). Furthermore, we revealed a highly significant loss of LTM memory retention in the strain with *CSE* deletion, which also suggests an important role of endogenous H_2_S in the cognitive processes. It is clear that there is a complex interaction between two major genes (*CBS* and *CSE*) playing a central role in cysteine metabolism and H_2_S production. Analysis of the number of genes that change the level of expression in strains with a single deletion of the *CBS* gene and strains with a double deletion revealed an interesting phenomenon: the number of genes with reduced expression level decreases almost three-fold in the strain with the double deletion as compared to the *CBS-/-* strain (Figure 5). This phenomenon should have a positive effect on various physiological processes. Indeed, previously, we demonstrated [67] to our surprise that the strains with double deletions exhibited significantly better stress tolerance and higher longevity than strains with a single *CBS* deletion.

It is likely the learning and memory failure observed in the strains with *CBS* deletion stems from a cumulative effect of simultaneous down-regulation of several pertinent genes involved in the cognitive processes in Drosophila males and the deleterious effect of *Hcy* accumulation (Figure 7).

It is well-known that molecular mechanisms underlying learning, memory formation, and long-term memory retention are quite different [68]. Thus, learning and short-term memory do not require the synthesis of new proteins and are formed by modifications of existing target proteins in the neurons responsible for cell membrane conductance and neuronal excitation [69,70].

In the case of learning and STM formation, the main processes include adenylate cyclase activation, cAMP production, and protein kinase A (PKA) activation. These processes include PKA-dependent phosphorylation of various proteins, including potassium and calcium channel subunits, resulting in the strengthening of pre-existing synaptic connections [67]. Therefore, the initial level of expression of both PKA itself and the genes related to potassium and calcium channel functioning may affect the formation of short-term memory. Consistent with this, our transcriptome analysis revealed a decrease in the expression of the following genes that may be responsible for the observed STM and learning disorders in strains with *CBS* deletions, and especially in the case of double deletions: *pka-c1*, *eag*, *Sh*, and *cac*. The ether a go-go gene (*eag*) participates in voltage-gated potassium channel activity and is involved in learning [71]. Gene Shaker (*Sh*), encoding the structural alpha subunit of a voltage-gated potassium channel, plays a key role in maintaining electrical excitability in neurons regulating neurotransmitter release at the synapses [72]. The cacophony (*cac*) gene is of special interest for the understanding of our results [73,74]. Like *Sh*, it encodes the structural subunit of the voltage-gated calcium channel located in presynaptic active zones and is involved in the release of neurotransmitters. The expression of this gene is essential for a wide range of neurophysiological processes; in particular, it contributes to male courtship behavior. Interestingly, *D. melanogaster* strains with deletions of 4–6 copies of the *hsp70* gene are also characterized by impaired memory formation processes, and exhibited a decrease in the expression level of the above mentioned genes [35].

In contrast to STM, long-term memory requires a synthesis of new proteins and the formation of new synapses [67,75]. During the formation of LTM, there is a persistent increase in the level of cAMP, phosphorylation of the transcription factor CREB (CRE-binding protein), and induction of CREB-induced gene transcription [68,76]. Surprisingly, strains with *CBS* and *CSE* deletions showed no changes in the expression level of the *creb* gene. It is known that LTM formation and long-term potentiation (LTP) are associated with enhanced neurotransmitter release and strengthening of synaptic connections [77,78]. A postsynaptic increase in calcium concentration is observed in most synapses that support LTP. Increased calcium levels lead to activation of Calcium/calmodulin-dependent protein kinase II (CaMKII) which is regulated by the Ca^2+^/calmodulin complex and autophosphorylation process [62]. Therefore, CaMKII is a key protein kinase involved in neural plasticity and memory formation [79,80,81,82]. Experimental blocking of CaMKII inhibits synaptic transmission [83]. Accordingly, in our studies, we observed a decrease in the expression level of the *CaMKII* gene predominantly in the strains with double deletions and strains with *CBS* deletion. This observation is consistent with the evidence demonstrating that, in mammals, H_2_S promotes long-term potentiation and regulates intracellular calcium concentration in brain cells. It has also been shown that H_2_S enhances LTP in synapses, and Ca^2+^ waves are induced in the surrounding astrocytes [84,85]. These facts corroborated the conclusion postulating that H_2_S may act as a neuromodulator and/or intracellular messenger and play an important role in synapse remodeling [85].

It is also well-established, that the regulation of RNA transport and the regulation of translation of localized mRNA play a very important role in synaptic remodeling [86]. Characteristically, in our studies, we observed down-regulation of several genes (*appl*, *ago1*, *orb2*, *elav*, and *cam*) belonging to this category in all strains with *CBS* deletion. In addition, the genes controlling the neuronal signaling and neurotransmission, as well as genes involved in neurotransmitter transport *cpx*, *Syt1*, *Syt7*, *unc-13*, *cac*, *Vap33*, and *Syn*, are down-regulated in the strains with deletion of *CBS* (Figure 7).

Analysis of genes, down-regulated in all strains with deletions, revealed genes with serine hydrolase activity or serine endopeptidase activity (Tables S1 and S2). Serine hydrolases have been shown to regulate proteolysis at the synapses and alter neuronal plasticity [87]. These genes also participate in posttranslational modification of key brain signaling proteins [88,89] and may affect the metabolism of a wide range of chemical messengers, including neurotransmitters [90,91,92]. It is possible that the altered expression level of genes involved in proteolysis may also contribute to the observed lack of long-term memory consolidation observed in the *CSE-/-* strain. We hypothesize that complete memory loss in flies with *CBS* gene deletion was caused by the cumulative effect of homocysteine accumulation and the lack of neuroprotective and neuromodulatory effects of H_2_S.

## 5. Concluding Remarks

In the experiments performed in this study, we used several *D. melanogaster* strains developed by the CRISPR/Cas9 technique containing double and single deletions of two major genes (*CBS* and *CSE*) responsible for H_2_S production. These strains with deletions have been investigated in experiments controlling learning and memory formation in flies using the mating rejection paradigm. The performed experiments demonstrated that all strains containing a single or double deletion of *CBS* were characterized by the complete block of long- (LTM) and short-term (STM) memory formation. Characteristically, flies with *CSE* deletion exhibited normal STM and LTM, but failed to exhibit LTM retention in our behavioral experiments. Transcriptomic studies have shown that deletions of the *CBS* and *CSE* genes significantly alter genome expression in male fly heads to varying degrees, with the *CBS* gene deletion having a more pronounced effect. Importantly, the genes with altered expression predominantly belong to the down-regulated group and include genes involved in learning and memory, reproductive behavior, cognition, and the oxidation-reduction process. Strains with double deletions exhibited the maximal degree of downregulation of the above-mentioned genes, while in the flies with the deletion of *CSE* only, we observed a very slight, hardly detectable decrease in the expression of the same groups of genes. The accumulated data enables us to conclude that neurotransmitter H_2_S plays an important role in learning and memory processes in the fruit flies by interacting with definite groups of genes and signal systems.

## Figures and Tables

**Table 1:** The number of genes with altered expression in the strains with deletions after pairwise comparison to control strain 548492 (FDR < 0.05).

*Category*	Number of Genes	*Category*	Number of Genes
*CBS-/-* genes down-regulated	295	*CBS-/-* genes up-regulated	72
*CBS-/-*; *CSE-/-* genes down-regulated	190	*CBS-/-*; *CSE-/-* genes up-regulated	61
*CSE-/-* genes down-regulated	53	*CSE-/-* genes up- regulated	20

## Data Availability

Sequence data were deposited in the NCBI GEO database under the number—GSE200397.

## Supplementary Materials

The following supporting information can be downloaded at: https://www.mdpi.com/article/10.3390/biom12060751/s1, Figure S1. Olfactory behavior assay results; Figure S2. Validation of *MST* level by quantitative RT-PCR in the heads of 5-day-old naïve males. Figure S3. Quantitative analysis of methionine/homocysteine and cystathionine amino acid levels in control strain, *CBS-/-*, *CSE-/-*, and double deletion strain *CBS-/-*; *CSE-/-*. Figure S4. KEGG pathways; Figure S5. Validation of *GBA1b*, *Teq*, *Ago1*, and *rut* gene expression levels by quantitative RT-PCR in the heads of 5-day-old naïve males. Figure S6. Graphical illustration of interrelations between differentially expressed genes (blue) and corresponding GeneOntology terms, involved in memory formation (purple) in a network of (*CBS-/-*; *CSE-/-*(1)) strain. Table S1. Primers for Q RT-PCR. Table S2. Representation of differentially expressed genes (down-regulated) from Figure 5A.; Table S3. Representation of differentially expressed genes (up-regulated) from Figure 5A.
