# Supplementary material for: Genes Responsible for H2S Production and Metabolism Are Involved in Learning and Memory in Drosophila melanogaster
Source: Biomolecules. 2022 May 26;12(6):751. doi: 10.3390/biom12060751 (PMC9221306; doi:10.3390/biom12060751)
Supplement: Supplementary file 1 [file biomolecules-12-00751-s001.zip › biomolecules-1703449-SI.pdf]

Supplementary Materials

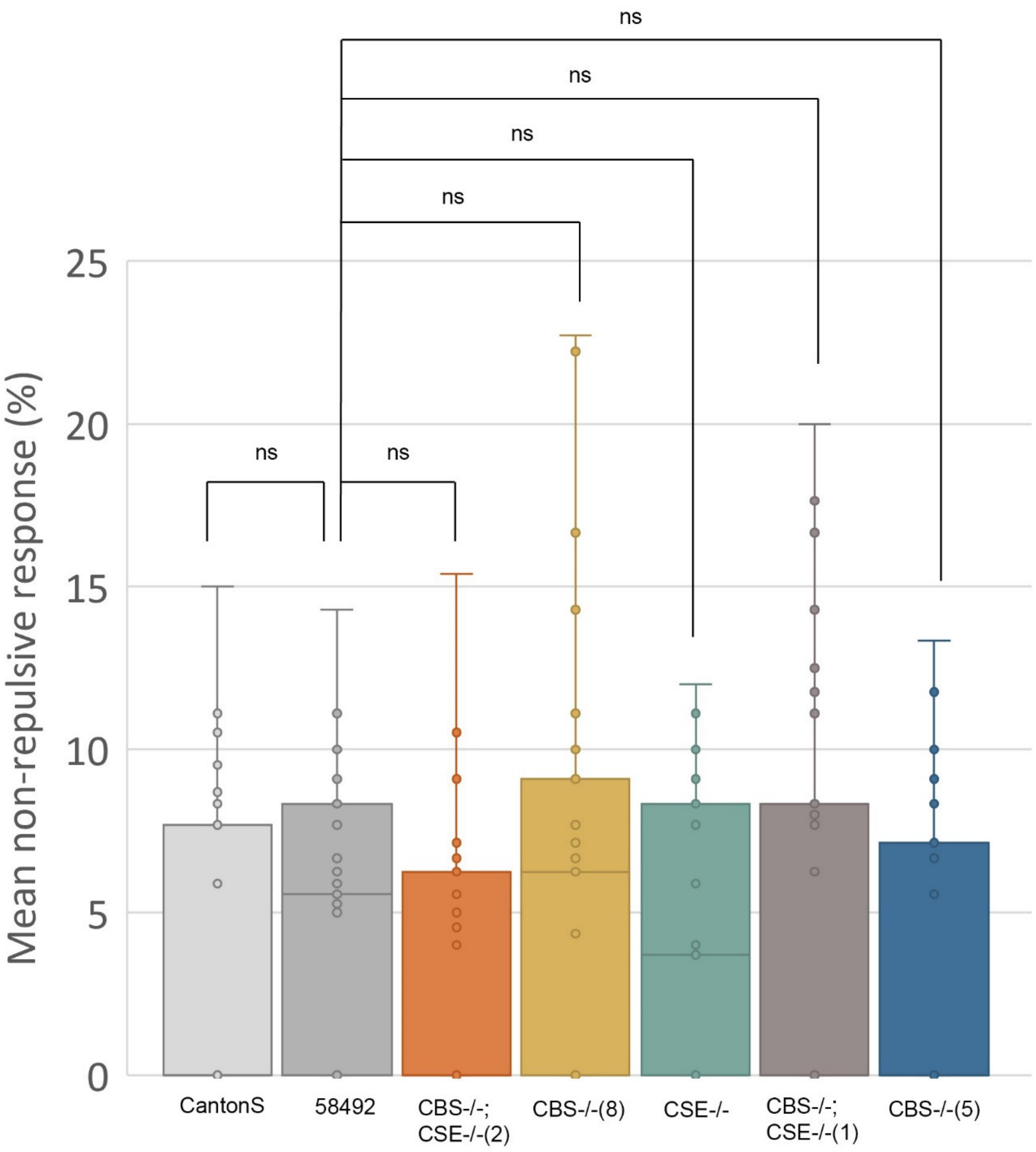

Figure S1. Olfactory behavior assay results. The percentage of flies demonstrating non-repulsive behavior to 4-methyl cyclohexanol. Mann–Whitney test, $p > 0.05$, ns – no significant difference, N = 400.

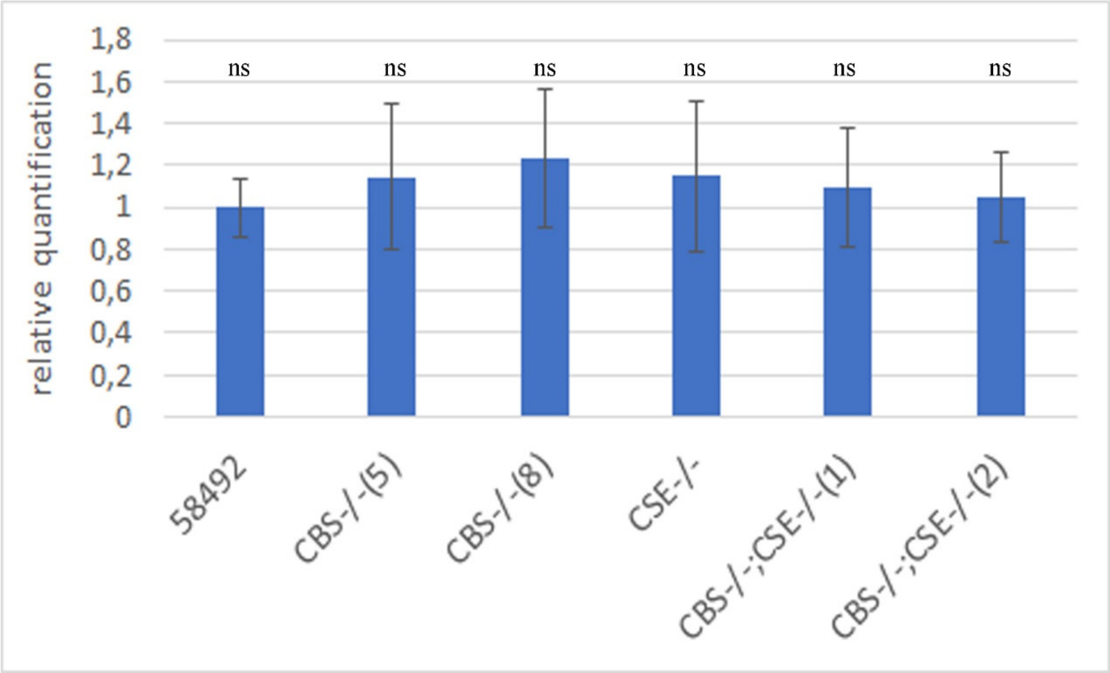

Figure S2. Validation of *MST* level by quantitative RT-PCR in the heads of 5 day old naïve males. The *MST* mRNA level was measured by quantitative RT-PCR and normalized to *rp49*. Each value represents the average with a standard deviation from three independent experiments. Each value represents the average with a standard deviation from three independent experiments. Ns - p> 0.05 (Students *t*-test).). In each experiment, a pool of 70 flies was collected.

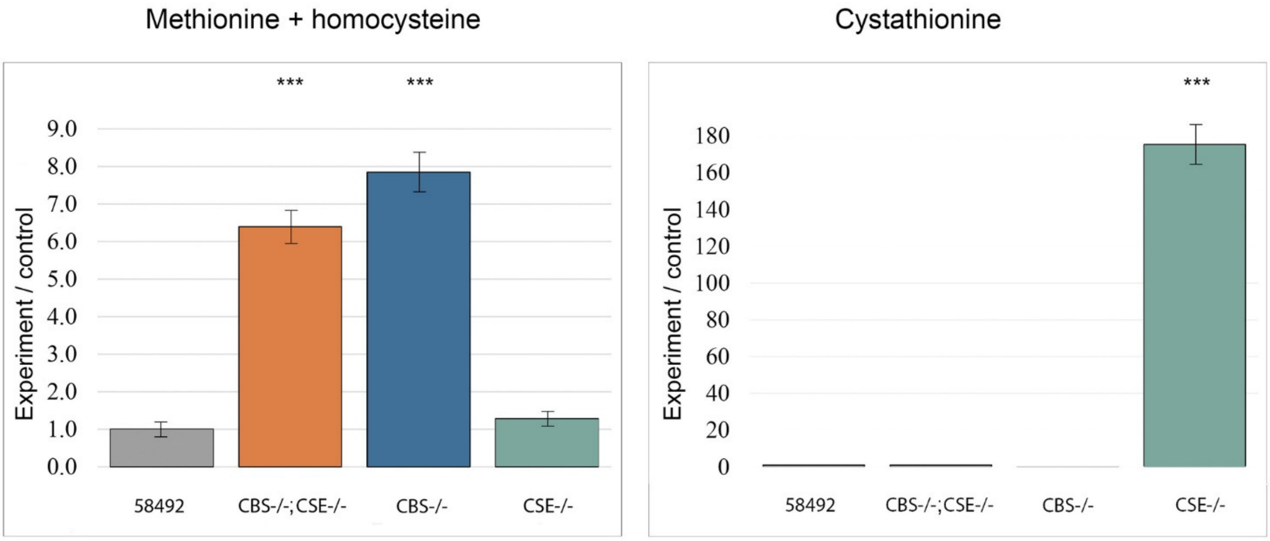

Figure S3. Quantitative analysis of methionine/homocysteine and cystathionine amino acid levels in control strain, CBS-/-, CSE-/- and double deletion strain CBS-/-, CSE-/-. The amount of amino acids is represented as a ratio of the content of the amino acids (percentage of the total content) in the experiment and control (mean ± SEM).

Data were obtained by summing the results of three independent experiments. ***- significant differences when compared to the value for the same amino acid in the control (P < 0.001).

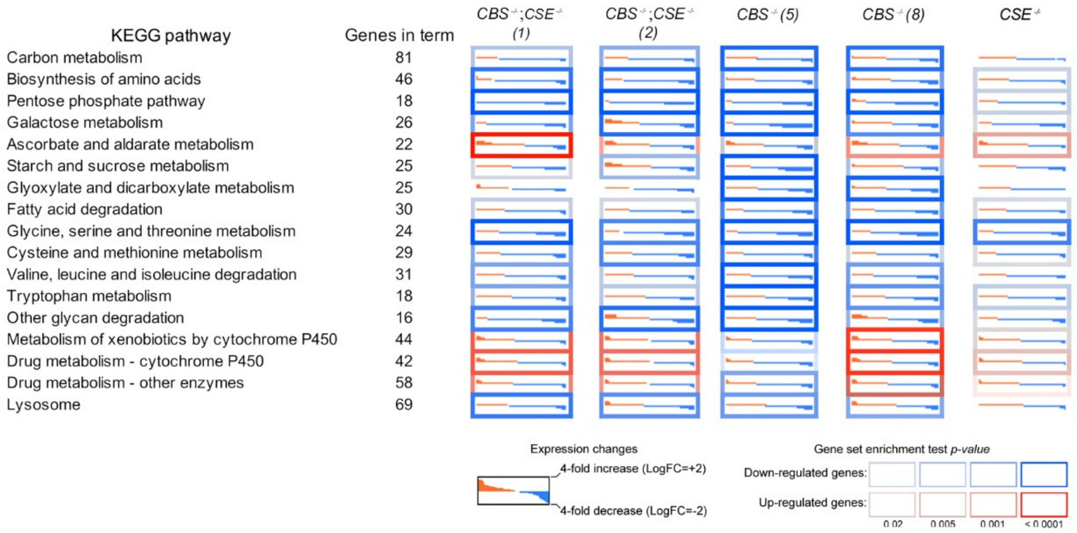

Figure S4. KEGG pathways. Differential expression profiles of genes participating in the most affected KEGG pathway deletions. The effects of single deletions of *CBS, CSE,* and double deletion (*CBS*-/-, *CSE*-/-) genes in male heads are depicted. Red and blue subplots illustrate expression level changes (log-scale; values are sorted in each subplot) between strains with deletions and control strains. The log expression level fold change (LogFC) range is from -2 (i.e., four-fold downregulation; blue) to ±2 (i.e., fourfold overexpression; red). Cell borders indicate the gene set enrichment (Fisher's exact test) *p*-value for a pathway. The red border indicates that a KEGG pathway is enriched with upregulated genes; the blue border indicates downregulated genes. *min. p* (*up/down reg*) – minimal gene set enrichment test (Fisher's exact test) *p*-value for up/downregulated genes across all four presented analyses. (Color figure online). (For interpretation of the references to color in this figure legend, the reader is referred to the Web version of this article.).

The performed KEGG analysis of transcriptomic data from *Drosophila* heads (Figure. S3) revealed rather similar results to the transcriptomic data from whole flies, but more pronounced changes in several metabolic pathways in *CBS*-/- genotype strains. In the strains with the *CSE* deletion, the changes are significantly less significant. In the case of fly heads in contrast to whole flies data, we observe additionally up-regulation of such pathways as ascorbate and aldarate metabolism, retinol and porphyrin metabolism, and down-regulation of lysosome pathway. Besides, we revealed the downregulation of several pathways related to the biosynthesis of amino acids and their degradation and carbohydrate metabolism

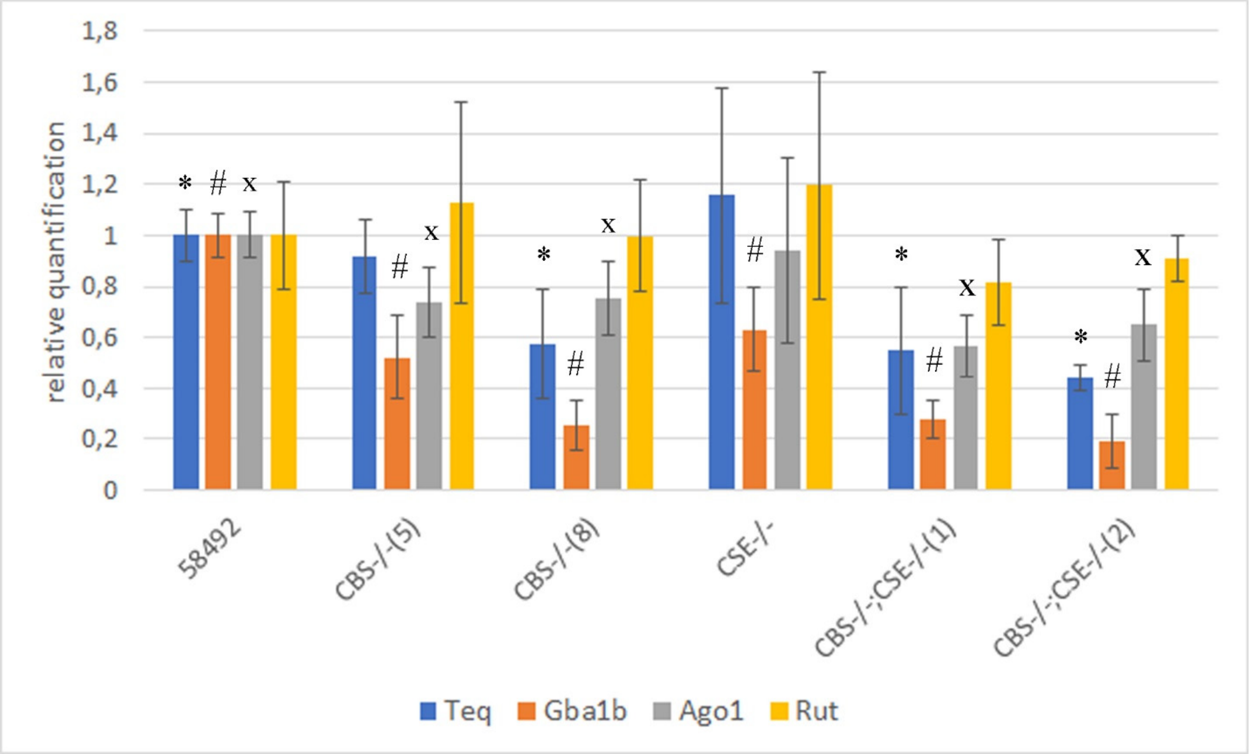

Figure S5. Validation of *GBA1b*, *Teq*, *Ago1*, and *rut* gene expression levels by quantitative RT-PCR in the heads of 5 day old naïve males. *GBA1b*, *Teq*, *Ago1*, and *rut* mRNA levels were measured by quantitative RT-PCR and normalized to *rp49*. Each value represents the average with a standard deviation from three independent experiments. *, #, x - $p < 0.05$ (Students *t*-test). In each experiment, a pool of 70 flies was collected.

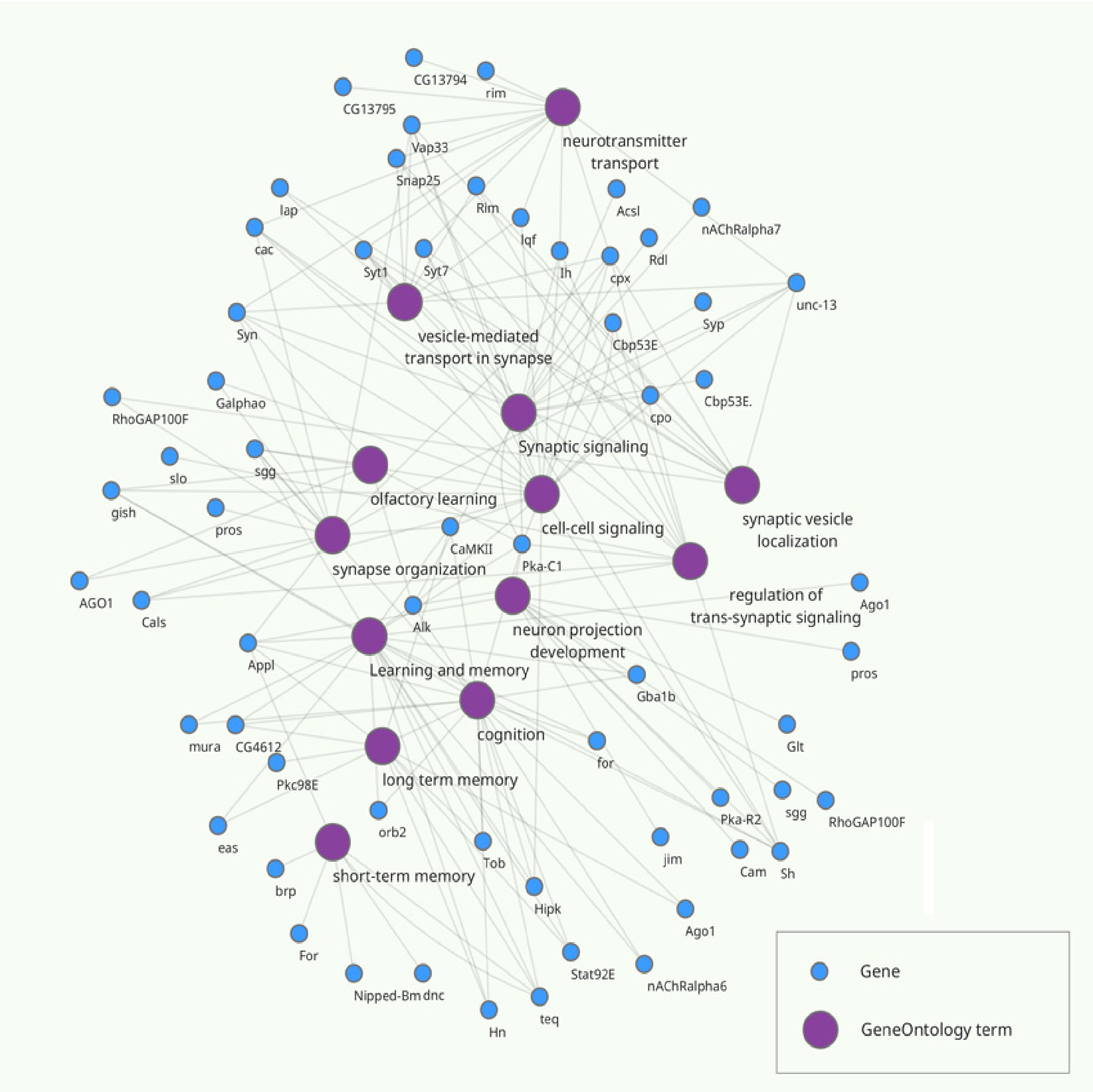

Figure S6. Graphical illustration of interrelations between differentially-expressed genes (blue) and corresponding GeneOntology terms, involved in memory formation (purple) in a network of *CBS−/−; CSE−/−(1) strain*. The visualization was performed using Cytoscape software.

Table S1. Primers for Q RT-PCR used in the study.

Primers	Sequence
**MST F**	5’-GGAGGAGGACTTCGCCCAG-3’
**MST R**	5’-ATCGGACGCAGTTGGGGTG-3’
**rp49 F**	5’-ATGCTAAGCTGTCGCACAAAT-3’
**rp49 R**	5’-GTTCGATCCGTAACCGATGT-3’
**Gba1b F**	5’-CTATCTGGCTGAACGACAATCT-3’
**Gba1b R**	5’-GCCATCCAAGTAGTTCAGCGA-3’
**Teq F**	5’-CCTTTTGATGACTTGGGCTGT-3’

**Teq R**	5’-CAGCCGACGTGAAGGAGGA-3’
**Ago1 F**	5’-CCTCGCAGTGGAAGATGATG-3’
**Ago1 R**	5’-CGGGGCGGCGAGTGACG-3’
**Rut F**	5’-GCTCTGGTCTTCGTCCTCTG-3’
**Rut R**	5’-GTGGGCAGGTGGTGGTCCTT-3’

Table S2. Representation of differentially expressed genes (down-regulated) from Figure 5A. (Fdr < 0.05). Gene groups common to: 1- all deletions *CBS*, *CSE*, and (*CBS-/-*, *CSE -/-*); 2- *CBS* and *CSE*; 3- *CBS* and (*CBS-/-*, *CSE -/-*); 4- *CSE* and (*CBS-/-*, *CSE -/-*); 5- unique for *CBS*, *CSE*, (*CBS-/-*, *CSE -/-*) are presented. For each gene FlyBase information is given. The table uses the abbreviation for (*CBS-/-*) - CBS, (*CSE -/-*) - CSE, double deletion strains (*CBS-/-*, *CSE-/-*) - BE.

Table S3. Representation of differentially expressed genes (up-regulated) from Figure 5A. (Fdr < 0.05). Gene groups common to: 1- all deletions *CBS*, *CSE*, and (*CBS-/-*, *CSE -/-*); 2- *CBS* and *CSE*; 3- *CBS* and (*CBS-/-*, *CSE -/-*); 4- *CSE* and (*CBS-/-*, *CSE -/-*); 5- unique for *CBS*, *CSE*, (*CBS-/-*, *CSE -/-*) are presented. For each gene FlyBase information is given. The table uses the abbreviation for (*CBS-/-*) - CBS, (*CSE -/-*) - CSE, double deletion strains (*CBS-/-*, *CSE-/-*) - BE.
